# Lipopeptide adjuvants for antibiotics and vaccines: the future step in the fight against multidrug-resistant and extensively drug-resistant pathogens

**DOI:** 10.37349/eds.2024.00043

**Published:** 2024-04-29

**Authors:** Chloé O. Sebilleau, Steven J. Sucheck

**Affiliations:** Department of Chemistry and Biochemistry, University of Toledo, Toledo, OH 43606, USA

**Keywords:** Lipopeptide, adjuvant, vaccine, antibiotic

## Abstract

With the surge of antibiotic resistance in bacteria, the need for a larger arsenal of effective antibiotics and vaccines has drastically increased in the past decades. Antibiotics like vaccines can benefit from significant potentiation when used in combination with adjuvants. Antibiotic adjuvants can allow for gram-positive bacteria (GPB) specific treatments to be used against gram-negative bacteria (GNB) infections, with minimal antimicrobial resistance (AMR). In the case of vaccines, they allow for modulation and increase of the immune response. Lipopeptides are molecules of choice because of their ability to activate specific cell surface receptors, penetrate the outer membrane of GNB, safety and ease of synthesis. This review explores the recent developments in lipopeptide adjuvants for antibiotics and vaccines, providing a roadmap on how to develop adjuvants to efficiently combat AMR. After a brief overview of bacterial resistance, lipopeptide adjuvants for antibiotics and vaccines are discussed, providing insights into stability, sources, and delivery methods. Findings discussed in this review could be applied to the development of safer, more effective adjuvants, that could expand the use or repurpose current antibiotics or improve vaccination results in future clinical trials.

## Introduction

This review explores the potential of lipopeptides as adjuvants for both antibiotic and vaccine development. Antimicrobial resistance (AMR) is currently one of the greatest public health threats associated with 5 million deaths in 2019 and up to 10 million deaths annually by 2050, representing a cost of 100 trillion US dollars (USD) [1]. Since the discovery of penicillin by Fleming in 1928, numerous other antibiotics have been approved for human use and bacteria, whether gram-negative bacteria (GNB) or gram-positive bacteria (GPB) have developed resistance against them. With the limited development of new classes of antibiotics in the last decades [2], the means of fighting multidrug-resistant (MDR) and extensively drug-resistant (XDR) pathogens are decreasing.

### What are lipopeptides?

The potential of low molecular weight lipopeptides, comprised of a peptidyl moiety, linear, branched, or cyclic, bound to a lipid chain will be explored in this review. Lipopeptides mostly originate from bacteria and fungi and are involved in pathogenicity, communication, and competition for nutrients. These amphiphilic molecules have the ability to cross the outer membrane of GNB thanks to their hydrophilic peptide chains as well as the plasma membrane thanks to their lipophilic chains, and therefore potentiate existing antibiotics. They can also make good vaccine adjuvants because they are able to activate specific receptors on host cells that trigger downstream immune responses.

### Potentiation of antibiotics by lipopeptides

Antibiotics can be potentiated to increase their range of applications. GPB specific antibiotics can be repurposed and used against GNB, and AMR against the existing antibiotics can be slowed down [3–5]: using an antibiotic in combination with an adjuvant allows for resistance to occur at a significantly slower rate than when the antibiotic is used without an adjuvant. This could be particularly useful as new antibiotics are not being developed fast enough to cope with the prevalence of MDR and XDR pathogens. Current antibiotic adjuvant pairs include Augmentin, a commercially available combination of amoxicillin and clavulanate. This drug is regularly used to treat β-lactamase producing pathogens that usually cannot be treated by penicillin or its derivatives, such as amoxicillin [6, 7]. Resistance to Augmentin is less prevalent than resistance to amoxicillin [8], a trend that can also be seen in other combinations of antibiotics and adjuvants. Amongst the different adjuvants that can be used to increase the activity of existing antibiotics, lipopeptides are a target of choice, as they can increase outer membrane permeability and affect the efflux pump machinery of GNB while being relatively simple and inexpensive to synthetize. Antibiotics are especially hard to develop for GNB because they must get through both membranes to get to their target while being non-toxic for humans. This is particularly difficult as bacteria adapt to survive even in conditions with high concentrations of antibiotics, by upregulating efflux pump expression, sequestering the antibiotic target, and modifying or destroying the antibiotic itself by enzymatic processes, amongst others [9]. Developing a new antibiotic is also not cost-effective. It’s estimated that from conception to the Food and Drug Administration (FDA) approval of a new antibiotic, approximately 1.5 billion USD is spent. However, it would only generate a revenue of 46 million USD per year based on average costs and sales, with an exclusivity period of around 10 years [10]. With the latest new class of antibiotics having been discovered in the 1980s, developing adjuvants rather than antibiotics could be a better solution to provide cost-effective treatments for MDR and XDR bacterial infection.

### Lipopeptides as vaccine adjuvants

Resistance to vaccines is significantly less prevalent than resistance against antibiotics [11]. Vaccines are the reason for the low occurrence of diseases caused by bacterial infections such as whooping cough, tetanus, and diphtheria and can decrease the number of occurrences where antibiotics are necessary. However, the development of new vaccines is challenging. Whole-cell vaccines although very immunogenic, however, can elicit local adverse reactions like in the case of whole-cell *pertussis* (wP) vaccines which are being replaced by subunit acellular *pertussis* (aP) vaccines, conferring less protection [12]. The efficacy of the vaccines is partly attributed to the type of immune response each of the vaccines offers: wP gives a balanced type 1 T helper cell (Th1)/Th17, required for optimal protection [12, 13], whereas aP gives a Th2 response because of the alum adjuvant required for immunogenicity [14]. The traditional alum adjuvant is not sufficient for the variety of immune responses required to obtain protection against various pathogens like *Pseudomonas aeruginosa* (*P. aeruginosa*) amongst others [15]. Although other adjuvants have been approved by the FDA, such as saponin fraction 21 of *Quillaja Saponaria* bark extract (QS-21), cytosine phosphoguanine (CpG), and monophosphoryl lipid A (MPL) [16], there is an urgent need for novelty in this field. Lipopeptides are a promising class of molecules in this field as they are immunomodulatory, can stimulate pattern recognition receptors (PRRs) on the surface of antigen-presenting cell (APC), and can be used for intranasal vaccination.

This review will provide insights into recent developments in antibiotic and vaccine lipopeptide adjuvants. Other adjuvants for antibiotics [4, 5, 17] and vaccines [16, 18, 19] have been extensively reviewed recently and will not be discussed here. Higher molecular weight lipoproteins will not be discussed here. The first part of this review will give a brief overview of the mechanisms of antibacterial resistance. The second part will be focused on the development of lipopeptide adjuvants for antibiotics and explaining their action mechanism, addressing concerns about their hemolytic and cytotoxic activity as well as providing some solutions to increase their *in vivo* stability. The third part will present promising advances in the field of lipopeptide adjuvants for vaccines, and discuss the improvement of their stability *in vivo*, potency, and their application in the field of intranasal vaccination.

## Bacterial resistance

Mechanisms of resistance in GNB and GPB have been reviewed previously [20, 21] and will only be briefly described in this section. Resistance can be acquired, adaptive, or intrinsic (Table 1). Bacteria can exchange DNA material vertically to gain new resistance genes. This was the case with β-lactamase and metallo-β-lactamase production in both GNB and GPB, conferring them resistance to penicillins [22], the most commonly used antibiotics in the USA [23]. Biofilm formation can also limit the effect of antibiotics [24] as only a few can disrupt it and expose bacteria. Biofilm can be formed by GNB such as *P. aeruginosa* [25] or GPB such as *Staphylococcus aureus* [26] and cause chronic infections as well as the growth of bacteria on catheters and prosthetics. The outer membrane plays an important role in GNB resistance, as the highly polar nature of lipopolysaccharide only allows small charged molecules to pass through it. Once in the periplasmic space, efflux pumps can cast out undesired material, such as antibiotics. The inner membrane of GNB only allows for lipophilic molecules to enter. Taken together those intrinsic barriers make GNB infections harder to treat. Adaptive resistance arises from prolonged exposure to increasing concentrations of antibiotics. Over a few generations, bacteria can upregulate their production of efflux pumps, increasing their resistance to antibiotics. Once removed from this environment, bacteria reverse back to their previous phenotypes in just a few generations [27, 28].

## Lipopeptide adjuvants for antibiotics

Lipopeptide adjuvants mostly function as efflux pump inhibitors or outer membrane permeabilizers and allow antibiotics that would not have reached the cytoplasm otherwise to act on their target. Antimicrobial peptides (AMPs) and lipopeptide antibiotics like polymyxin and colistin have been sources of inspiration for designing adjuvants. Synergistic and additive effects of antibiotics with lipopeptides and peptidomimetics are particularly interesting in the context of limited antibiotic discovery, as they can reduce the minimum inhibitory concentration (MIC) of antibiotics that do not usually have activity against GNB low enough to clear infections due to GNB. Synergistic effects will be discussed in terms of fraction inhibitory concentration index (FICI), with FICI values below 0.5 interpreted as synergy and FICI comprised between 0.5 and 4 interpreted as additive. All discussed lipopeptide adjuvants for antibiotics are summarized in Table 2.

### Adjuvants derived from lipopeptide antibiotics

**Figure 1.**
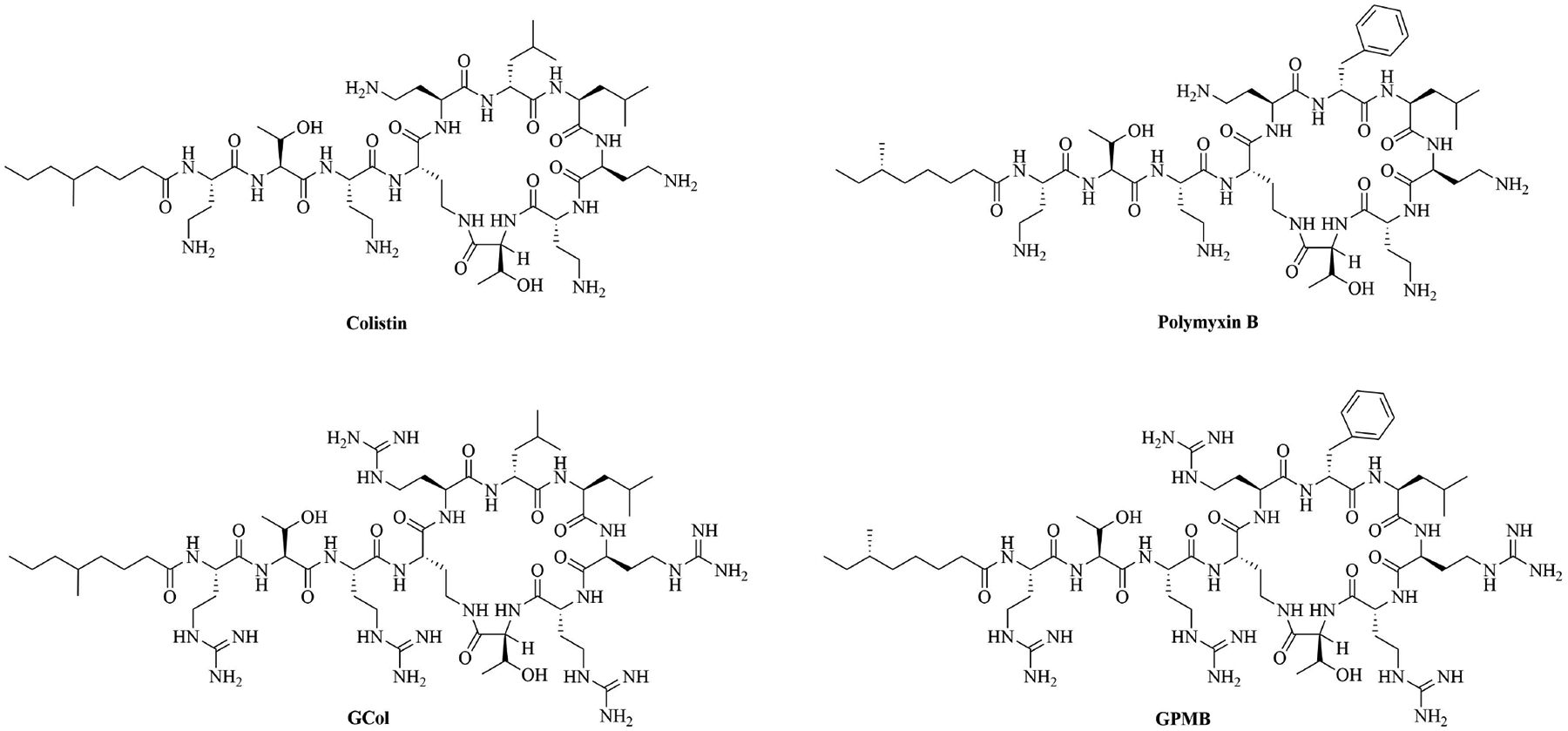
Structures of colistin, polymyxin B, GCol, and GPBM

Colistin and polymyxin (Figure 1) are cyclic lipopeptides traditionally used as last resort antibiotics against GNB infections [33]. Modification of polymyxin with guanidine decreased its antibiotic activity but increased its capacity to permeate the outer membrane. The combination of GCol and GPMB (Figure 1) with rifampicin, novobiocin, and erythromycin was synergistic against wild type *E. coli*, *A. baumannii*, and *P. aeruginosa* [34]. GPMB was able to synergize with vancomycin, clindamycin, and linezolid against the same bacteria. In particular, the combination of rifampicin and GCol had MICs in the range of 4 ng/mL to 63 ng/mL, and the combination of GPMB with rifampicin had MICs values in the range of 1 ng/mL to 63 ng/mL against colistin-susceptible and colistin-resistant *P. aeruginosa*, *E. coli* and *A. baumannii* [34]. Those membrane permeabilizers were not able to synergize with antibiotics that are also membrane permeabilizers. They were also more efficient against *P. aeruginosa* because the AMR of this bacteria mostly comes from its impermeable outer membrane than they were against *A. baumannii* which also strongly relies on efflux pumps for its AMR [34, 44].

### AMPs as a source of adjuvants

**Figure 2.**
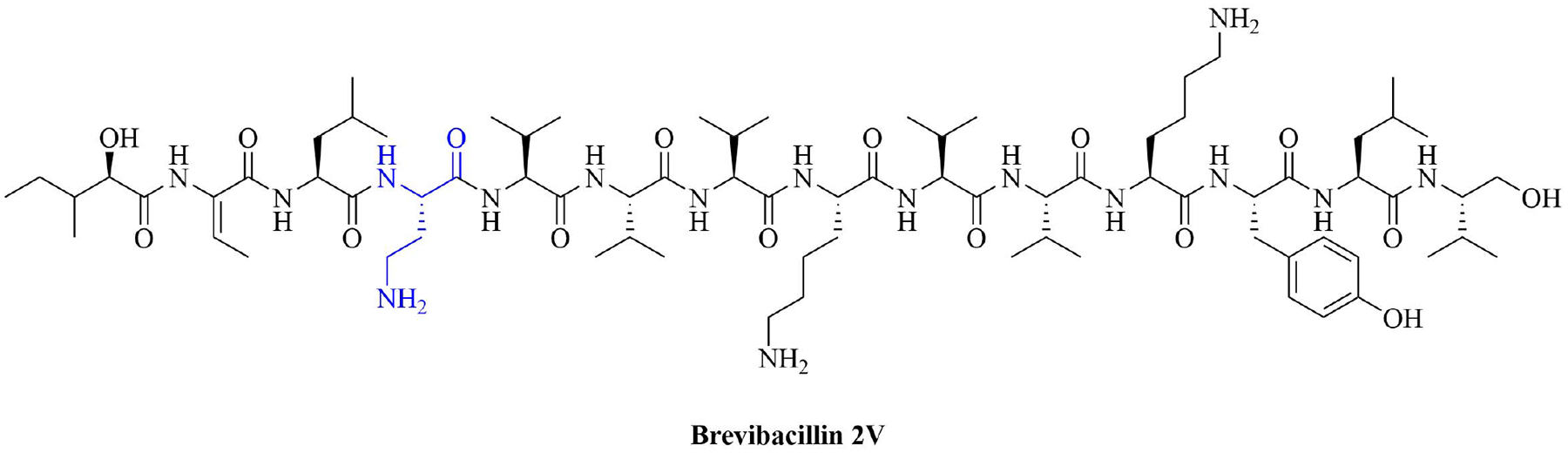
Structure of brevibacillin 2V. Non-canonical ornithine has been highlighted in blue

Bacteria secrete their own AMPs, but they are usually toxic to mammalian cells or are highly hemolytic. GPB *Brevibacillus laterosporus* secretes brevabacillins, which are AMPs and have activity against other GPB [35, 36]. These AMPs, especially brevabacillin 2V (Figure 2) can be used as adjuvants for increased potency of antibiotics traditionally used for treatment of GPB, against GNB. Specifically, brevabacillin 2V was used in combination with nalidixic acid, azithromycin, rifampicin, and amikacin against members of the ESKAPE family such as *E. coli*, *K. pneumoniae*, *P. aeruginosa*, and *A. baumannii* in a synergistic manner against all pathogens, reducing the MIC of those antibiotics up to 64-fold [35]. Furthermore, this lipopeptide has no hemolytic activity, low cytotoxicity as well as a high resistance to proteases. The stability in sera of this lipopeptide can be explained by the presence of non-canonical AAs in its peptide core [35].

### Peptidomimetics to increase stability to proteolysis

Peptidomimetics is a great way to increase the stability of proteases and potentiate antibiotics, as they can reach lower hemolytic and cytotoxic activity than AMPs while having an increased synergistic activity with current antibiotics. The following section will discuss recent lipopeptidomimectics with high synergistic activity in MDR and XDR strains of bacteria.

**Figure 3.**
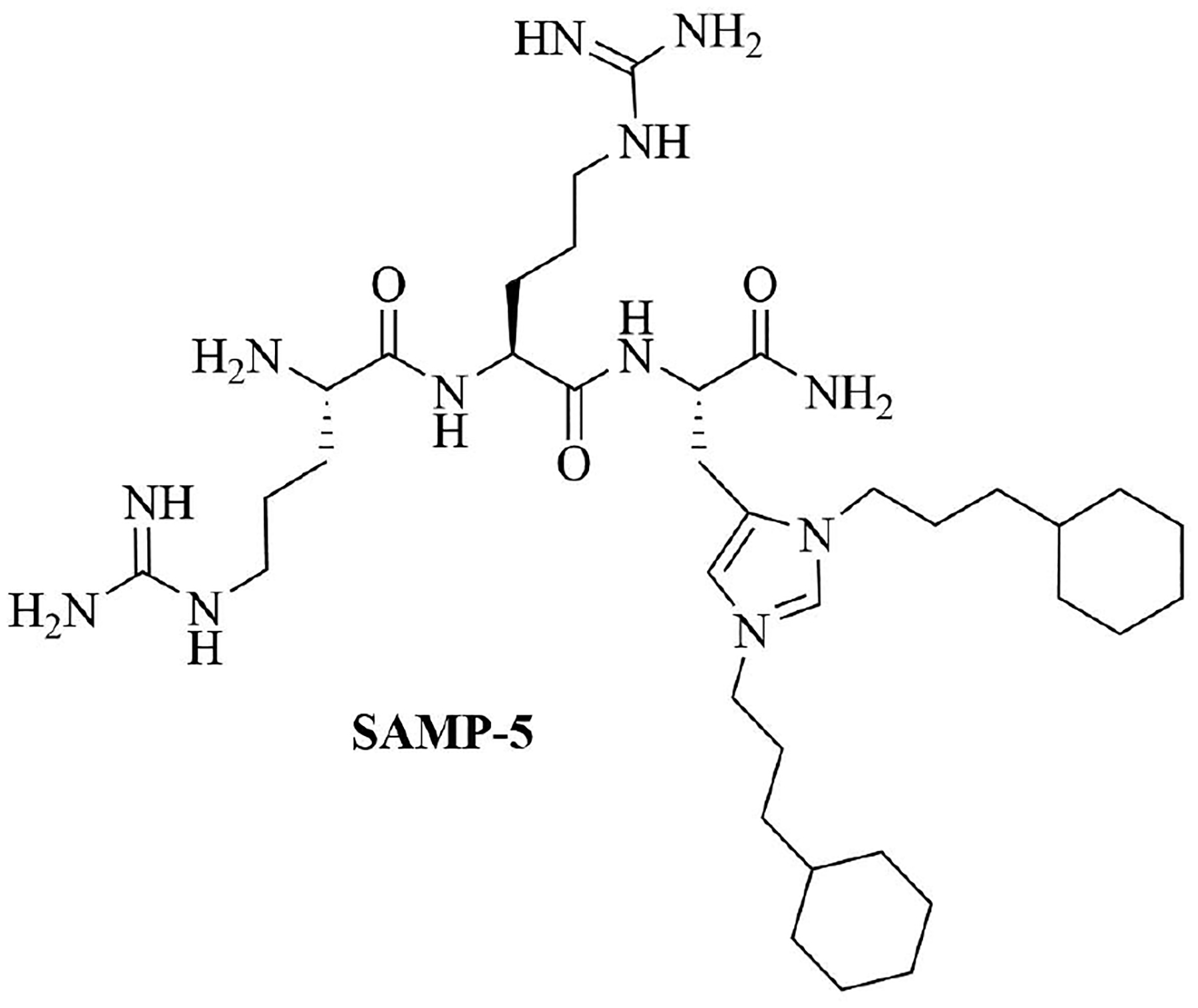
Structure of SAMP-5

SAMP-5 (Figure 3) has activity against MDR *E. coli* and *P. aeruginosa* (MIC between 16 μg/mL and 64 μg/mL) [37] and can also be used as an adjuvant with antibiotics such as ciprofloxacin, oxacillin, and chloramphenicol. In the case of MDR *P. aeruginosa*, synergy was observed with all three of the abovementioned antibiotics, especially in the case of ciprofloxacin (FICI = 0.0781). In the case of MDR *E. coli*, synergy was observed with oxacillin and ciprofloxacin with ciprofloxacin having the greater synergistic effect again (FICI = 0.0165). No resistance against SAMP-5 was observed after seven passages in *P. aeruginosa*, the MIC of SAMP-5 was not increased over those passages whereas the MIC of ciprofloxacin was increased 16-fold over four passages [38].

**Figure 4.**
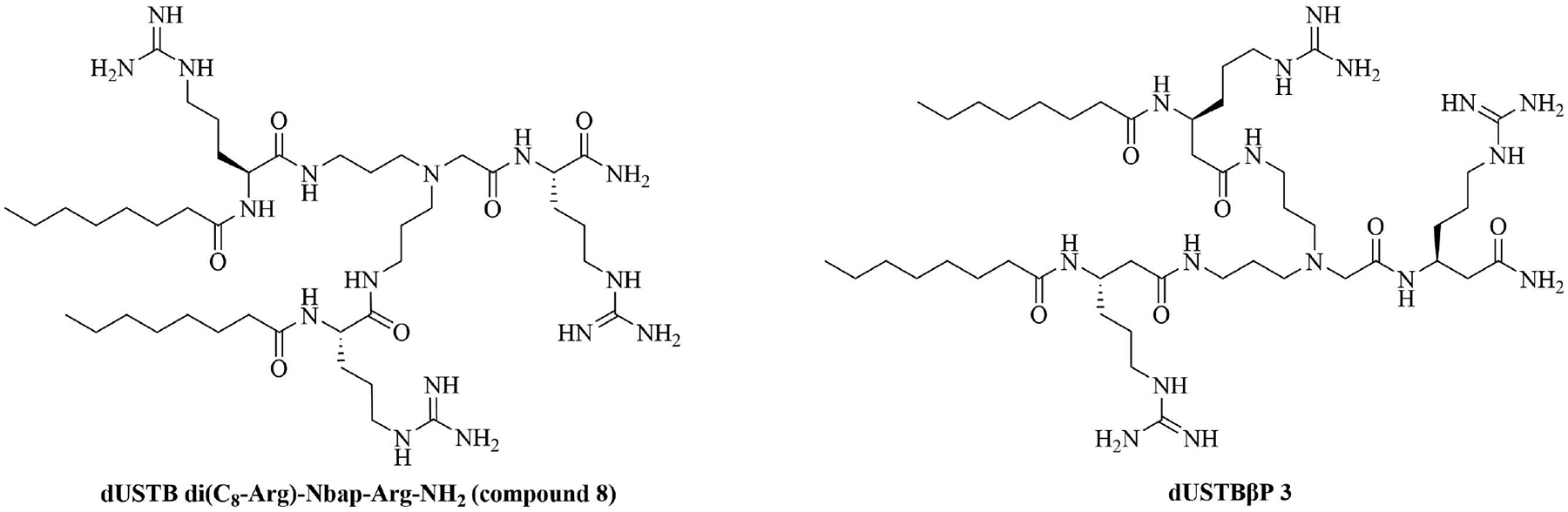
Structures of compound 8 and dUSTBβP 3

Dilipid ultrashort cationic peptides, containing only four AAs, have been an alternative to AMPs but are still sensitive to proteases. A strategy to increase in sera stability is to disrupt the peptidyl backbone, by incorporating β-AA or alkyl chains. These compounds, dUSTBPs have synergistic effects with traditional GPB antibiotics against GNB. Geometry of these compounds influences their stability and activity: branching in the scaffold leads to increased protease stability, whereas lipid chains of 12 carbons or more have an increased synergistic effect with current antibiotics but induce strong hemolysis. Compound 8 (Figure 4) has the best compromise between synergistic activity, by being able to synergize with novobiocin and rifampicin (FICI comprised between 0.004 and 0.375, potentiation up to 256-fold) against MDR clinical isolates of *P. aeruginosa*, *A. baumannii*, and *Enterobacteriaceae*, and low levels of hemolysis (around 3.5%) [39]. The introduction of methylation at the N-ζ position of the lysine (Lys) led to decreased potentiator activity, with the tertiary amines being the less efficient and the primary amines being the most efficient adjuvants [45]. dUSTBβP 3 (Figure 4), incorporating β-AA instead of α-AA was identified as a strong potentiator with either an additive or synergistic effect for multiple classes of antibiotics in *E. coli*, *A. baumannii*, and *P. aeruginosa*. dUSTBβP 3 is nonhemolytic, noncytotoxic, and resistant to proteolysis. This lipopeptide was able to reduce the MIC of novobiocin below its breakpoint in MDR strains of *A. baumannii*, *E. coli*, *K. pneumoniae*, and *E. cloacae* [40]. The same effect was observed when dUSTBβP 3 was used with rifampicin against most MDR strains of the abovementioned bacteria. However, changing the α-AA to β-AA led to slower bactericidal activity [40].

**Figure 5.**
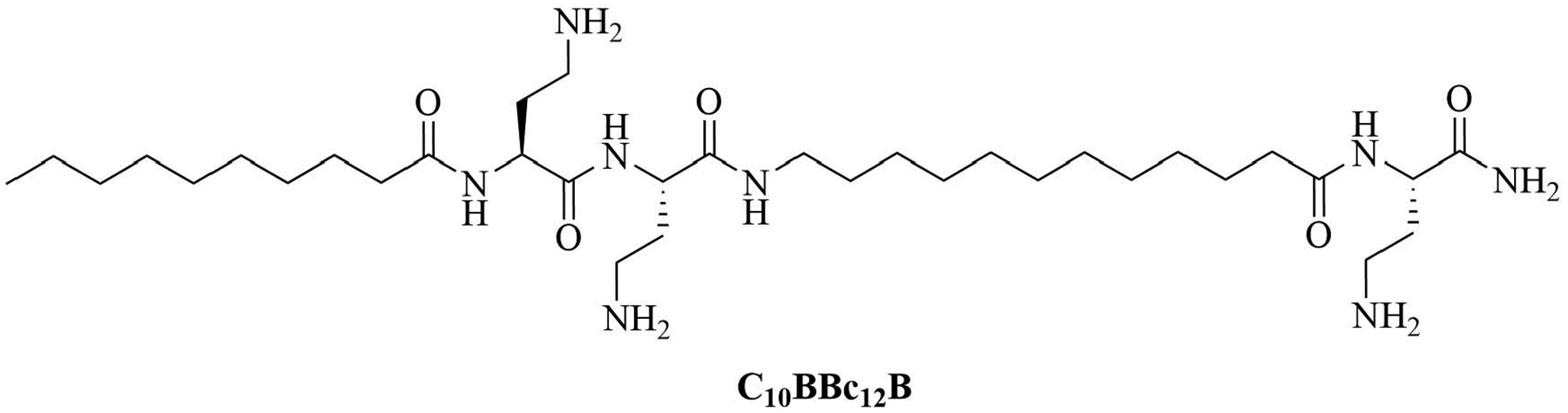
Structure of C_10_BBc_12_B

Lipopeptide like-pentamer C_10_BBc_12_B (Figure 5) was used in combination with erythromycin and rifampin, two antibiotics traditionally used to treat GPB infections but with less application in GNB infection treatments because of their low penetration of the outer membrane and showed synergistic activity against *E. coli* and *K. pneumoniae* with a FICI of 0.2. The inhibitory concentrations of erythromycin against *E. coli* dropped from 128 mg/mL to 0.125 mg/mL and against *K. pneumoniae* from 512 mg/mL to 0.250 mg/mL with the addition of C_10_BBc_12_B. In the case of rifampin, the MIC decreased 65,000-fold in the case of *E. coli* and 130,000-fold in the case of *K. pneumoniae*. *A. baumanii* elicited virtually no resistance to the combination of erythromycin and C_10_BBc_12_B (128-fold increase in MIC for erythromycin without C_10_BBc_12_B compared to 2-fold increase in MIC for the combination of the adjuvant and the antibiotic). Less than 2-fold increase in the MIC of rifampin in combination with C_10_BBc_12_B was observed compared to a 4-fold increase for the antibiotic on its own, proving that the use of adjuvants can increase the life span of antibiotics. The adjuvant antibiotic combination was tested *in vivo*: mice were lethally challenged with *E. coli*. Three days after the challenge, 80% of the control group mice died but 90% survived in the C_10_BBc_12_B erythromycin group [42]. The potency of this adjuvant could be attributed to its dual role as an outer membrane permeation agent as well as an efflux pump inhibitor, a role that also has been evaluated in similar compounds [41]. Adjuvants with similar structures have been used in combination with rifampin and showed high potency against GNB infections, increasing the survival rate from 20% to 60% in the case of a single dose of rifampin compared to a single dose of adjuvanted rifampin in mice challenged with *K. pneumoniae* [46].

### Encapsulation of antibiotics by lipopeptides

**Figure 6.**
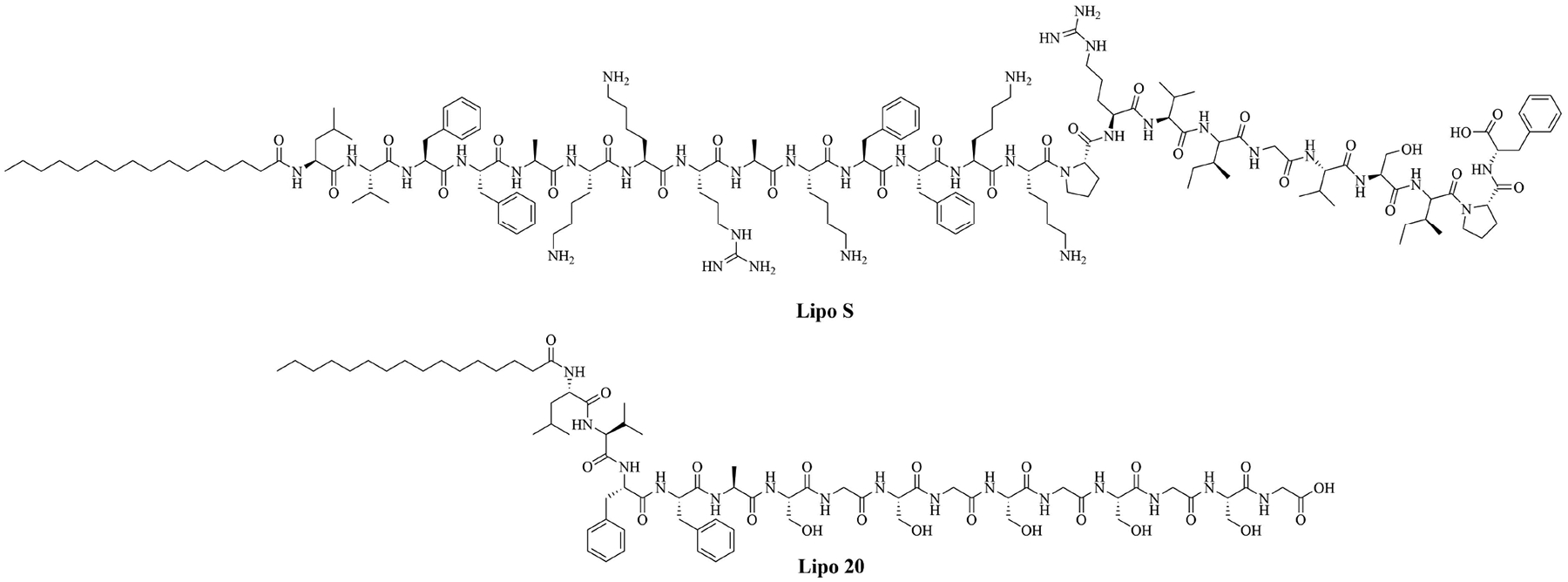
Structures of Lipo S and Lipo 20

Using lipopeptides to encapsulate antibiotics can increase their stability in sera and their cytoplasmic concentrations, leading to increased efficiency of the current antibiotics. Lipo S and Lipo 20 (Figure 6) were used to form self-assembling nano antibiotic transformers encapsulating antibiotics. In the case of *K. pneumoniae*, Lipo S and Lipo 20 were used to encapsulate ciprofloxacin. The self-assembled structures were spherical particles that transformed into long fibrils upon interaction with the bacterial cell wall, increasing the penetration of the antibiotic cargo into the cell. This transformation of shape did not happen when the nanoparticles were exposed to mammalian cells. The combination of the lipid nanoparticles and ciprofloxacin was synergistic, with a FICI of 0.3 and no resistance to the combinatorial drug appeared, even after 1 month. Lethal infection of *K. pneumoniae* killed all the mice that only received ciprofloxacin but all mice which received injection of the encapsulated antibiotic survived. This adjuvanted antibiotic also has low toxicity and hemolytic activity [43].

Lipopeptides are versatile compounds that can be used as membrane permeabilizers, efflux pump inhibitors, and self-adjuvanting delivery systems. Using them in combination with antibiotics has led to less resistance compared to using the antibiotic alone, which ensures longer days to the current antibiotics. They can also be used to repurpose drugs such as rifampin, used in GPB but not GNB infections, to broaden their scope and give even more treatment options to patients. Clinical trials still have to take place before any of these combinations can be used in humans, but murine infection models are encouraging, especially given the low hemolytic activity of those combinations and their little to no cytotoxic activity.

## Lipopeptides as vaccine adjuvants

Vaccines, unlike antibiotics, have little to no resistance against them [11] and can limit the occurrence of MDR and XDR strains by decreasing the number of infections due to bacteria in general, non-resistant, single drug-resistant, MDR or XDR, which will lead to less adaptations of the bacteria, and less resistance overall, but also by limiting the use or misuse of antibiotics by limiting the number of infections and therefore the need of treatment [47]. For this purpose, adjuvants based on pathogen-associated molecular patterns (PAMPs) have been developed. PAMPs are recognized by PRRs located on the surface, in the cytosol, or in the endosome of professional APC, amongst others. Adjuvants can take advantage of the receptors on APC to increase antigen cross presentation, upregulate cytokine production, and modulate the type of immune response obtained. One of the most studied PRRs is Toll-like receptors (TLRs). There are 10 different human TLRs: TLR1, TLR2, TLR4, TLR5, TLR6, and TLR10 are located on the cell surface of APC while TLR3, TLR7, TLR8, and TLR9 are located in intracellular compartments where TLR10 can also be found. TLR9 is the receptor of CpG but other TLRs such as TLR2 have been extensively used for adjuvanting purposes. TLR2 signals through the myeloid differentiation factor-88 (MyD88) pathway [48], leading to dendritic cell (DC) maturation and efficient antigen presentation. The role of TLR ligands and other PRRs in immunity has been nicely reviewed previously and will not be discussed in depth in this review article. TLR2 ligands include lipopeptides containing two or three lipid chains and a cationic peptide, but small molecules such as diprovocim have recently been identified as TLR2 agonists [49]. Extensive reviews of the role of adjuvants as TLRs [18, 50] have been published previously and this subject will not be discussed further here. All lipopeptide adjuvants for the vaccine discussed in this review are summarized in Table 3.

Lipopeptide adjuvants, include amongst others: macrophage-activating lipopeptide-2 (MALP-2), the first discovered TLR2 ligand, isolated from *Mycoplasma fermentas*, and its synthetic analogs, *N*-palmitoyl-*S-*[2,3-bis(palmitoyloxy)propyl]cysteinyl-seryl(lysyl)_3_-Lys (Pam_3_CSK_4_) and *S*-[2,3-bis(palmitoyloxy) propyl]cysteinyl-seryl(lysyl)_3_-Lys (Pam_2_CSK_4_) that have been extensively used for murine vaccination with intraperitoneal, intramuscular and intranasal delivery. They can self-assemble into nanoparticles, which can be endocytosed or phagocytosed if their size is around 10 μm to 500 μm [51]. Pam_3_CSK_4_ and Pam_2_CSK_4_ (respectively TLR2/TLR1 heterodimer and TLR2/TLR6 heterodimer agonists) are the most used lipopeptide adjuvants for vaccination and can elicit Th1, Th17, and Th2 immune responses. The stability of Pam_2_CSK_4_ and Pam_3_CSK_4_ in physiological conditions has been one of the biggest drawbacks of the use of those adjuvants in FDA-approved vaccines, as the rapid degradation of Pam_3_CSK_4_ in sera has been associated with decreased immune responses.

### Modifications of Pam_3_CSK_4_ and Pam_2_CSK_4_ to increase sera stability

Traditional lipopeptide adjuvants Pam_3_CSK_4_ and Pam_2_CSK_4_ have received a number of optimizations due to their poor stability in physiological conditions leading to weaker immune responses than expected. In particular, the ester bonds of Pam_3_CSK_4_ can be hydrolyzed and the serine of this lipopeptide can be oxidized in the serum [66]. Degradation of the adjuvant can be linked to decreased interaction with APC and downstream lower concentrations of antibodies. Modifications of Pam_3_CSK_4_ showed *in vitro* potency as TLR2/1 and TLR2/6 agonists [65–67].

**Figure 7.**
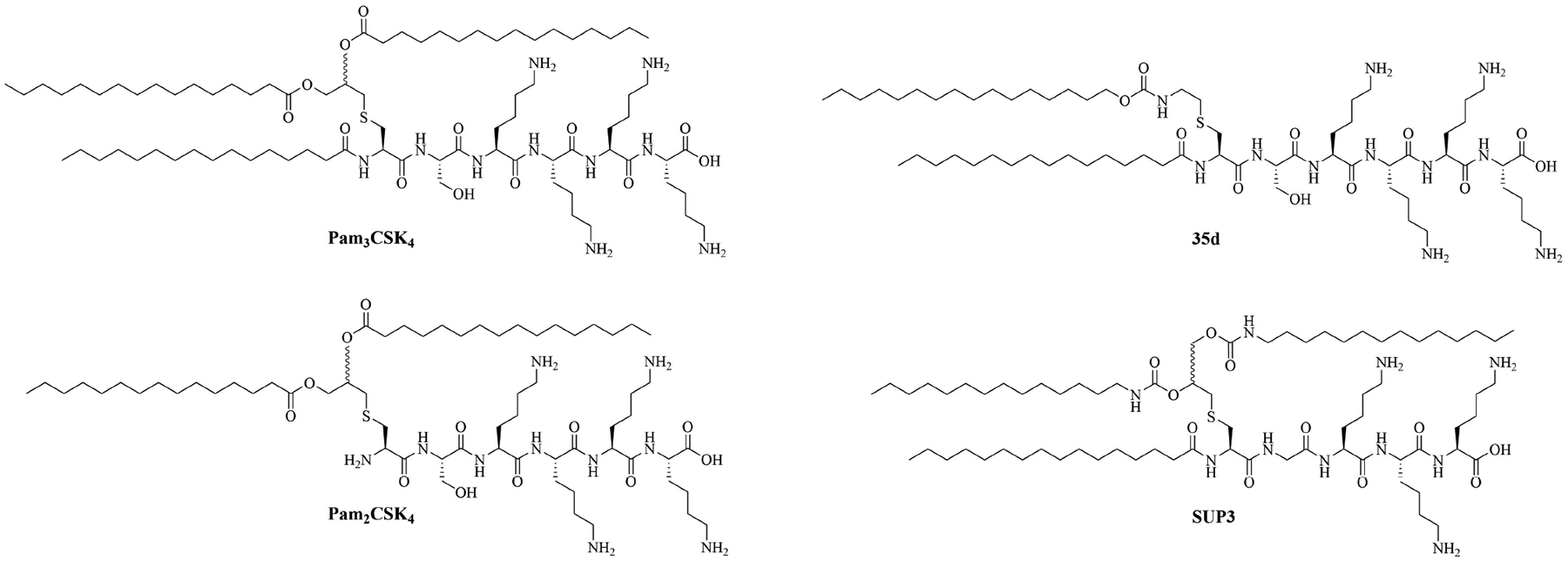
Structures of Pam_3_CSK_4_, Pam_2_CSK_4_, 35d, and SUP3

Compound 35d (Figure 7) has a similar potency to Pam_3_CSK_4_ [65] [upregulation of interleukin 6 (IL-6), tumor necrosis factor alpha (TNF-α), maturation of DCs through nuclear factor-kappaB (NF-κB) signaling pathway] and can activate both murine and human TLR2 [65]. Compound 35d features 2 lipid chains, one bound to the peptide moiety by a carbamate group (palmitoyl chain), the other one linked to a Lys by an amide bond (14 carbon atom long alkyl chain), limiting the degradation of the adjuvant in physiological conditions [65]. Similar modifications of the Pam_3_CSK_4_ scaffold have led to the development of SUP3 [66], a TLR2 agonist featuring three palmitoyl chains linked to a simplified peptidyl chain by two carbamates and one amide. In SUP3, the terminal Lys, which does not participate in hydrogen bonding in the TLR2 binding grove was removed and the serine was replaced by a glycine, eliminating the oxidizable hydroxyl group and suppressing one stereocenter. Those modifications afford a lighter, more stable TLR2 specific adjuvant. SUP3 has been tested with a tumor model but has not yet been used in a bacterial vaccine, like 35d, but show promising results and could potentiate higher immune responses than traditional Pam_3_CSK_4_ due to their increased stability.

**Figure 8.**
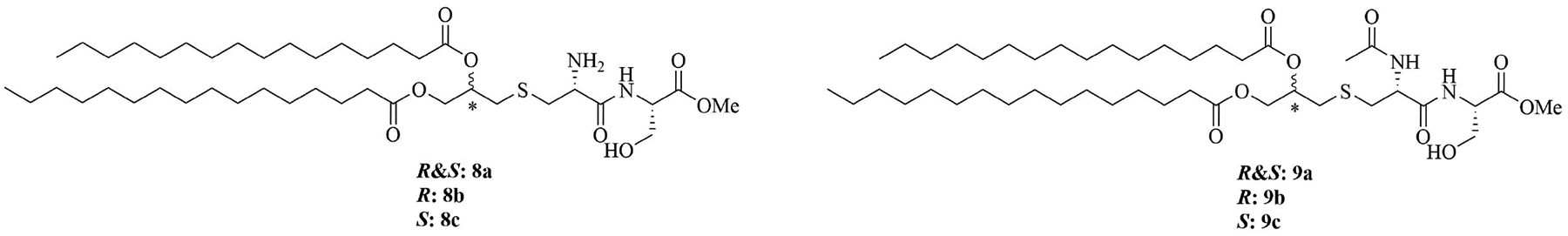
Structure of series 8 and 9

Structural optimizations of Pam_2_CSK_4_ showed that the glycerol moiety attached to the cysteine and the two lipid chains of Pam_2_CSK_4_ were essential for the TLR2 activity, as well as the presence of a peptide chain. The importance of the AA in the peptide chain is not clear as multiple peptides bearing the Pam_2_CS moiety have TLR activity and the four Lys are not required for activity [82]. Simplification of the Pam_2_CSK_4_ scaffold revealed two potent analog series (Figure 8): Pam_2_CS(OMe) and NAc-Pam_2_CS(OMe) [67]. For both series, the *R* isomers (8b and 9b) showed similar TLR2/TLR6 activity as per their half maximal effective concentration (EC_50_) values in human TLR2/6 reporter gene assay. The racemic mixture elicited almost comparable activity, even though slightly lower than the *R* isomers. The activity of the *S* isomers on the other hand was decreased 334-time for the Pam_2_CS(OMe) series and 315-time for the NAc-Pam_2_CS(OMe) series compared to their respective *R* isomers. Human peripheral blood mononuclear cells [DCs, monocytes, natural killer (NK) cells, T cells, and B cells] were able to secrete cytokines IL-6, IL-10, and TNF-α upon stimulation with series 8 and 9 to a higher concentration than Pam_2_CSK_4_. Compounds 8b and 8c were able to significantly upregulate the expression of clusters of differentiation 40 (CD40) in monocytes. Pam_2_CS (OMe) has been used in combination with a TLR7 agonist in nanoparticles for the vaccination of mice against the influenza virus and has shown promising results [68] but has not yet been used in anti-bacterial vaccines.

**Figure 9.**
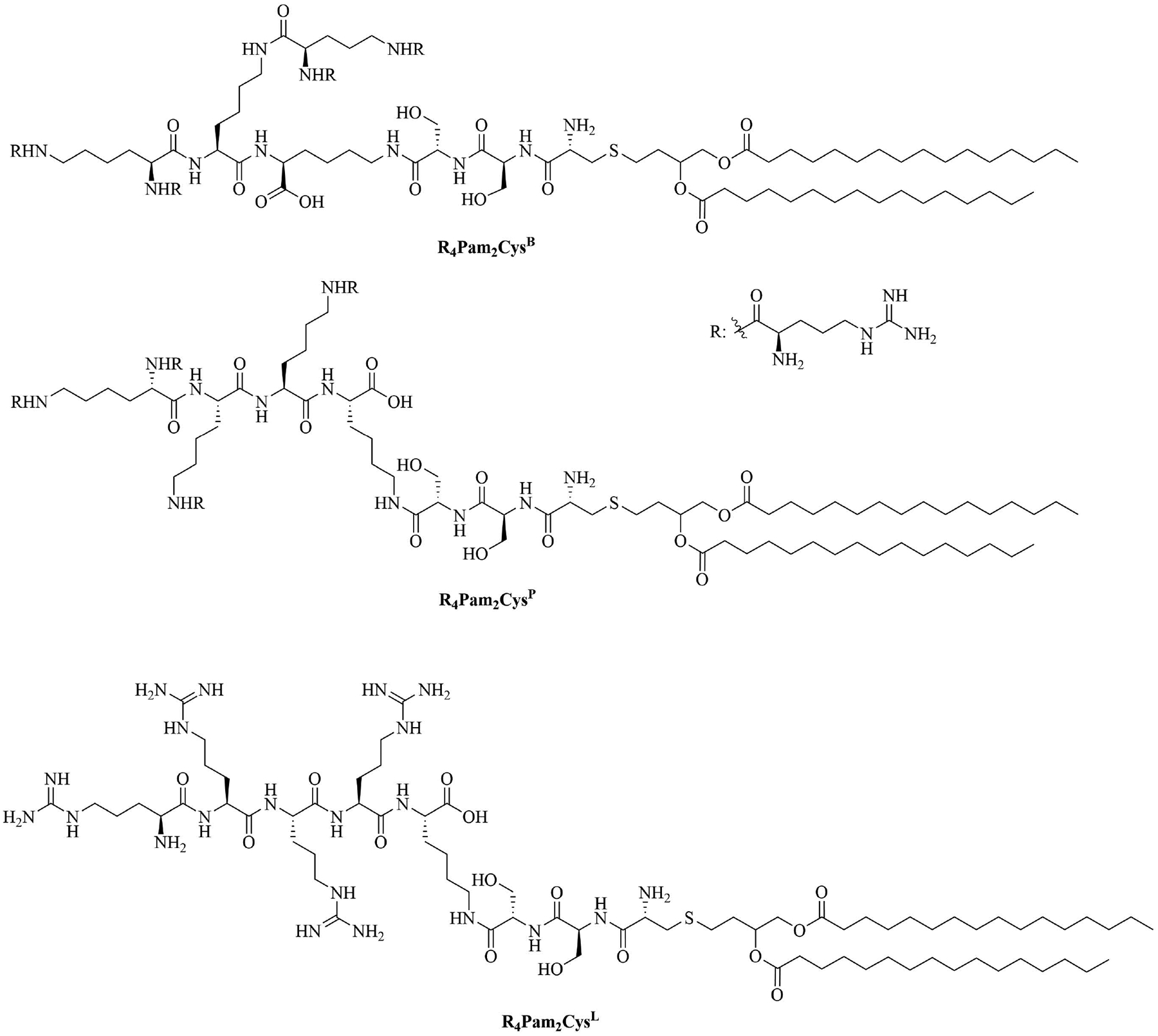
Structures of R_4_Pam_2_Cys^B^, R_4_Pam_2_Cys^P^ and R_4_Pam_2_Cys^L^

The peptide chain of Pam_2_CSK_4_ has been modified to improve stability and antigen presentation by introducing branching the CSK_4_ peptide chain was replaced by four arginine residues to form multiple isomers of R_4_Pam_2_Cys [69]. Positively charged R_4_Pam_2_Cys electrostatically bound to OVA, forming nanoparticles of around 500 to 600 nm hydrodynamic radius. To assess the *in vivo* activity of R_4_Pam_2_Cys^L^, R_4_Pam_2_Cys^B^, and R_4_Pam_2_Cys^P^ (Figure 9), OVA in combination with either of the adjuvant was administered to mice, a negative control group was vaccinated with OVA alone. No significant difference in OVA specific antibody titers were observed in adjuvanted groups. R_4_Pam_2_Cys^B^ and R_4_Pam_2_Cys^P^ were drastically more stable than their linear analog in naive mouse sera, with R_4_Pam_2_Cys^L^ depleting by 63% over 18 h whereas R_4_Pam_2_Cys^B^ and R_4_Pam_2_Cys^P^ were not proteolyzed [69]. The branched R_4_Pam_2_Cys^B^ compounds overall elicited an increase in the production of antigen specific interferon-gamma (IFN-γ) secreting CD8^+^ T cells and OVA specific antibodies, which could partly be due to the increased stability of the branched compounds but also to their increased flexibility enabling electrostatic interactions with more antigens than the linear form. The higher electrostatic charge of R_4_Pam_2_Cys^B^ could also allow cell penetration of the antigens to occur via additional pathways than TLR2 into the APC which could further explain the increased immune response compared to that of the linear adjuvant [69]. R_4_Pam_2_Cys^B^ was subsequently used in a vaccine against *Mycobacterium ulcerans* proving that this adjuvant is responsible *in vivo* for high antibody titers [83].

### Intranasal vaccination with lipopeptide adjuvants

New adjuvants could also pave the way for intranasal vaccination, as alum formulations for intranasal delivery of antigens have only been used successfully in a handful of pre-clinical vaccines [84] because of their toxicity. The nasal associated lymphoid tissue (NALT) is very rich in immune cells and intranasal vaccination has been associated with better protection and longer lasted immune response. NALT also ages at a slower rate than other lymphoid tissues, therefore intranasal vaccination could also benefit the elderly, which immune systems are weaker. This subject has been reviewed in dept recently [85] and will not be further discussed here. Only a few vaccines with intranasal delivery are in use as of today and development in this area is direly needed. Intranasal vaccination is particularly important when the main route of infection is through the mucosal membrane.

**Figure 10.**
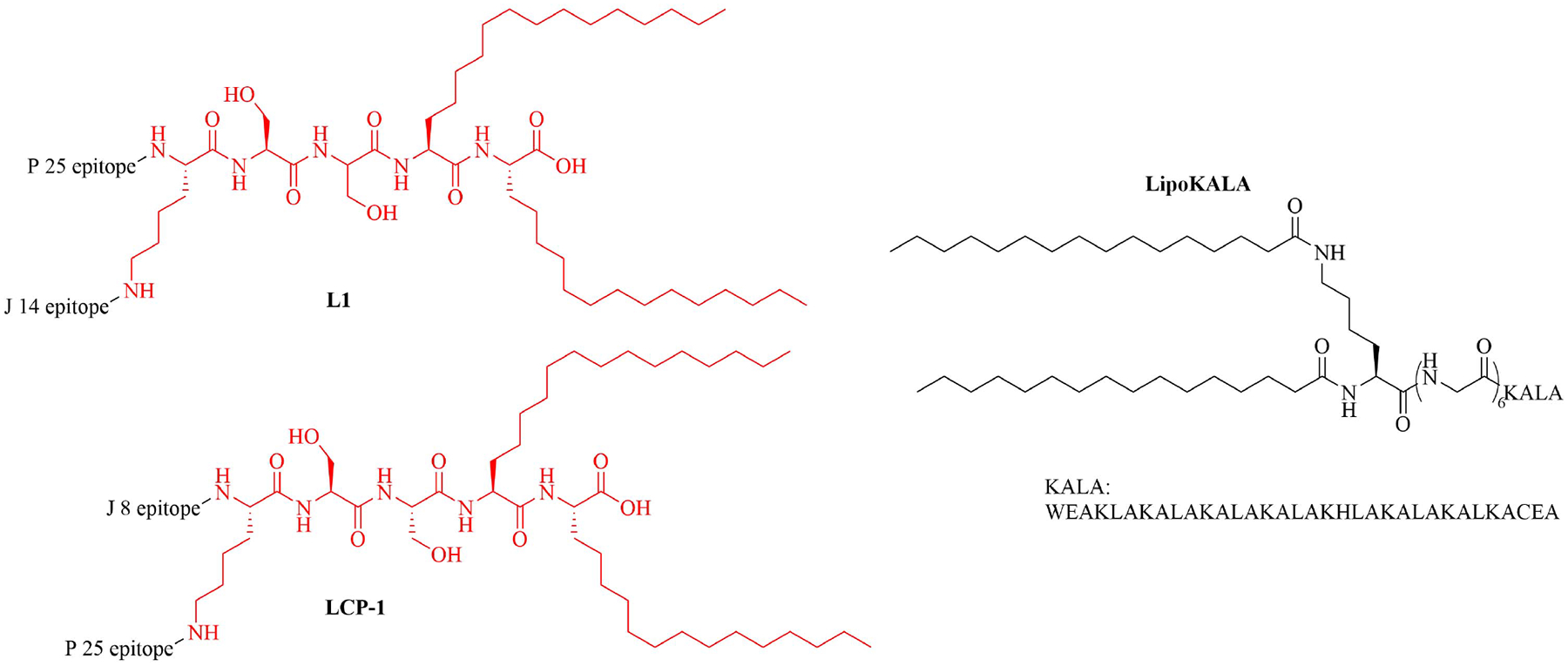
Structure of L1, LCP-1, and lipoKALA. In red: structure of LCP

The LCP motif, a TLR2 agonistic lipopeptide, has been successfully used for intranasal vaccinations as a self-adjuvanting antigen carrier. In the case of GAS vaccination, LCP was covalently fused to J14 and P25, a universal Th cell epitope. The construct was formulated into vaccines on its own (L1) (Figure 10) and in a cationic liposome formulation (L4) [70]. Five months after initial intranasal immunization, sera of mice vaccinated with L4 still had high titers of immunoglobulin G (IgG) and IgA. L4 was able to generate both systemic and mucosal anti-J14 antibodies. The liposome formulation also enabled the immune response to switch from an unbalanced Th2 response to a balanced Th1/Th2 response and to 6-time higher titers of IgA specific antibodies [70]. The increased immune responses in the presence of liposome could be due to an increase in antigen load, as multiple copies of the antigen are encapsulated inside the liposome, but also to an increased stability of the antigen, as it is not in direct contact with sera when encapsulated. The number of copies of the antigen delivered to the same APC also increases with the presence of liposomes [70]. Switching the liposome for nanoparticles of poly(lactic-co-glycolic acid) also yielded systematic and mucosal immunity, with adsorbed L1 being more efficient than encapsulated L1 [71]. The addition of cell penetrating peptides (CPPs) to liposomal formulations containing the J8 GAS epitope, as a replacement for the J14 epitope and P25 covalently linked to LCP (LCP-1) (Figure 10) drastically increased the immune response, especially in the case of lipidated CPPs. The introduction of lipidated CPP lipoKALA in the liposome formulation resulted in J8-specific opsonizing antibodies [72, 73].

Mucosal vaccination with lipopeptides has also been studied in the case of *Mycobacterium tuberculosis* (*M. tuberculosis*) [86, 87]. Early secreted antigenic target 6 kDa (ESAT-6) epitope was fused with Pam_2_CSK_4_ and the construct retained the TLR2 activity of Pam_2_CSK_4_. Mice were vaccinated intranasally and subcutaneously with the conjugate, using the *Bacillus Calmette-Guerin* (BCG) vaccine as a positive control. After three injections, the mice were challenged with a lethal dose of aerosol *M. tuberculosis* and the immune responses were assessed four weeks after the challenge. Intranasal vaccination of the mice resulted in a similar number of colonies in the lungs and spleen as vaccination with BCG. On the opposite hand, subcutaneous injection of the conjugate did not elicit nearly as strong protection [86, 87].

### Dual role of lipopeptides as carriers and adjuvants

Difficulties in the development of subunit vaccines can arise from choosing an appropriate carrier to increase immune potency. This is especially the case for carbohydrate vaccines, which cannot elicit specific immune responses in the absence of a carrier. Lipopeptides can be used as adjuvants and carriers, limiting the number of steps involved in the production process and simplifying the vaccine composition, leading to reduced costs.

LCP has been used as a carrier and adjuvant for carbohydrate subunit GAS vaccines as well as for intranasal delivery. The construct comprised of cell wall carbohydrates of GAS covalently linked to LCP self-assembled into nanostructures of sizes ranging between 300 nm and 500 nm and led to the production of opsonizing antibodies [88].

**Figure 11.**
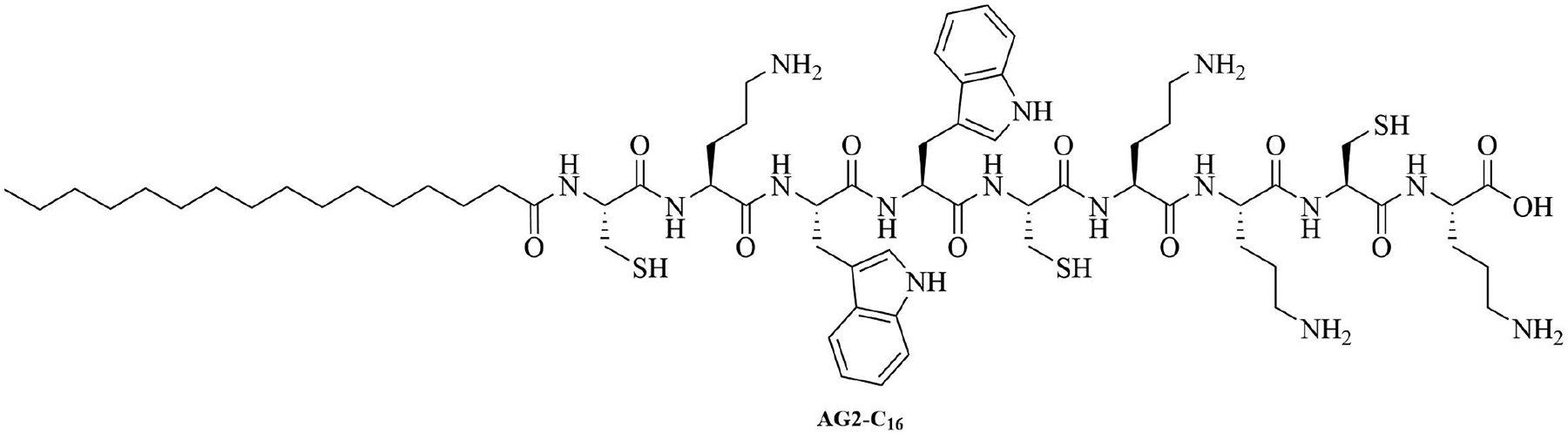
Structure of AG2-C_16_

Gemini lipopeptides can also be utilized for their dual role as carrier and adjuvant. The monomers are comprised of a lipid chain and a peptidyl chain that contains a cysteine, which can dimerize under physiological conditions to form Gemini lipopeptides. The immunogenicity of those dual carrier and adjuvants was assessed *in vivo* using BSA as a model antigen. AG2-C_16_ (Figure 11) was the most potent adjuvant and elicited a Th2 biased immune response in mice. The addition of CpG oligodeoxynucleotide (CpG-ODN) in the vaccine formulation further increased the immunostimulant effect of the Gemini compound, but vaccination with CpG-ODN as a single adjuvant did not lead to anti-BSA antibodies [74].

### Addition of another adjuvant leads to increased immunogenicity or change in the type of immune response

**Figure 12.**
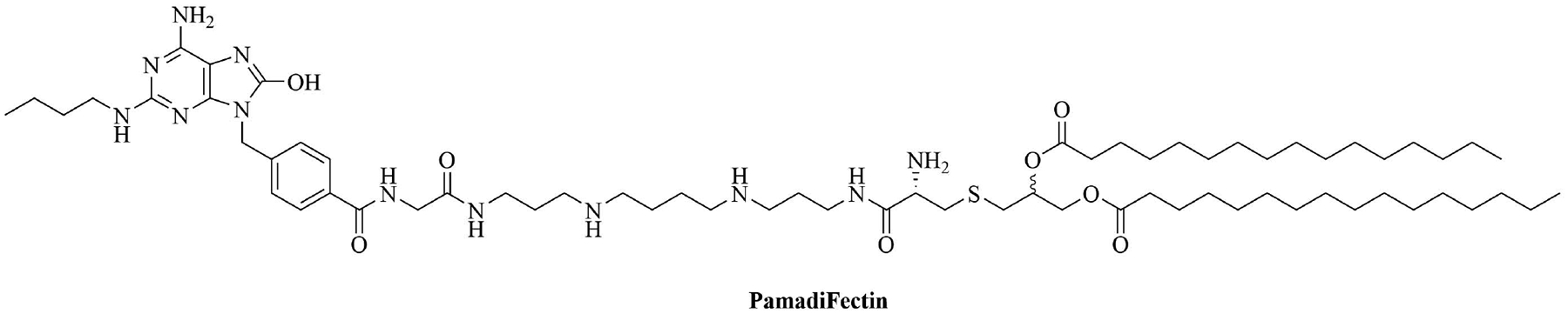
Structure of PamadiFectin

The combination of muramyl dipeptide (MDP), a nucleotide-binding oligomerization domain 2 (NOD2) agonist, covalently linked to a synthetically long peptide (SLP) based on OVA and Pam_3_CSK_4_ (abbreviated as Pam in this construct), MDP-SLP-Pam, preserved the TLR2 and NOD2 agonist effects of both the adjuvants [58]. TLR2-triggering was not enhanced by the synergy and kept the same levels as with the SLP-Pam construct but NOD2-triggering was enhanced compared to the MDP-SLP construct, which could be explained by the presence of the lipid moieties enabling easier penetration into APC. This construct mostly led to the synergistic secretion of pro-inflammatory cytokines, skewing the immune response towards Th1, instead of the Th2 response usually obtained from Pam_3_CSK_4_ adjuvanted vaccines. This could be due to the NOD2 and TLR2 pathway converging downstream and to NOD2 upregulating the expression of MyD88. The activation of TLR2 requires less agonist once NOD2 is activated. The activation of TLR2 and NOD2 can also be achieved with PamadiFectin (Figure 12), a dual TLR2/TLR7 adjuvant containing CL533 and Pam_2_CSK_4_ covalently linked by spermine groups, which has yet to be used for bacterial vaccination [75].

MDP was also lipidated to increase its potential as an immune adjuvant. MDP on its own was not able to elicit DC maturation but the addition of an *O*-stearyl ester at the C6 position leads to DC maturation. The addition of the lipid did not elicit any TLR2 activity [89].

Polyinosinic-polycytidylic acid (Poly I:C), a TLR3 agonist has been used in combination with Pam_3_CSK_4_. The mixture of the TLR agonists significantly upregulated the expression of CD80, CD86, CD25, and CD69 in B cells and synergistically increased the secretion of IL-6, TNF-α, C-X-C-motif chemokine ligand 10 (CXCL10) and IgG. Mice vaccinated with the combination of adjuvant and a protective antigen for anthrax once showed steady high levels of antigen specific antibodies even 20 weeks after injection, which was not observed in the mice vaccinated only with Pam_3_CSK_4_ and the antigen [60].

Pam_3_CSK_4_ has also been used in combination with polylactic acid (PLA) nanoparticles to create a pathogen-like biodegradable delivery system. Pam_3_CSK_4_ coats the surface of the PLA nanoparticles [90], forming spherical structures of around 150 nm in diameter, a size compatible with endocytosis. The coated nanoparticles retained the TLR2 activity of Pam_3_CSK_4_ in an *in vitro* HEK-Blue^™^-hTLR2 reporter assay, with 30% of Pam_3_CSK_4_ being available after 10 h. The 70% remaining could be released after endocytosis and the Pam_3_CSK_4_ molecules recycled to the surface of the cell to activate the TLR2 receptor again. PLA nanoparticles in combination with Pam_3_CSK_4_ were also used to entrap a TLR7 agonist. These studies [90] show that encapsulated TLR7 agonists can be released from the hydrophobic core of the nanoparticle over two days. Comparison between the nanoparticles containing only the TLR7 agonist and the TLR2 agonist showed that there was less TLR7 agonist release when the nanoparticle contained both adjuvants, suggesting that Pam_3_CSK_4_ not only was present on the surface of the nanoparticles but also to an extent encapsulated in them [91], corroborating with *in silico* observations [92]. This could prevent Pam_3_CSK_4_ from degrading in sera and increase its potential as an adjuvant without modifying its structure to include branching, reducing the complexity of synthesis.

Chitosan, a powerful activator of the NOD-like receptor family pyrin domain containing 3 (NLRP3) [93], was used in combination with Pam_3_CSK_4_ for vaccination of mice in the case of *Streptococcus mutans* (*S. mutans*) dental infection causing caries. Protein antigen c (PAc), a surface protein of *S. mutans* was used as the antigen in this study [59]. Vaccination of mice with Pam_3_CSK_4_, chitosan, and the antigen (PAc-chitosan-Pam_3_CSK_4_) elicited 3-fold higher antibody titers than PAc on its own. Upon challenge, all groups of mice developed caries but PAc-chitosan-Pam_3_CSK_4_ vaccinated mice had significantly more enamel volume than the PAc-Pam_3_CSK_4_ and less individual variability than the PAc-chitosan group [59].

Pam_3_CSK_4_ was modified to include a maleimide group, Pam_3_CSK_4_-Mal, which was coupled to a fusion protein of 4 protective epitopes of outer membrane porin F (OprF), an outer membrane porin of *P. aeruginosa*, by taking advantage of thiol maleimide coupling. The coupled protein was formulated into a liposome formed of dipalmitoylphosphatidylcholine and cholesterol. Upon vaccination, mice immunized with this vaccine formulation elicited a Th2 immune response, which is not as desirable as a Th1 immune response in the case of *P. aeruginosa*, as Th1 responses have been linked to better lung conditions in patients. QS-21 was added to the liposome formulation and mice immunized with this vaccine elicited a balanced Th1/Th2 immune response [54].

### Self-adjuvanted vaccines

Lipopeptides can be covalently attached to the antigen vaccine formulation, to increase the potency of the vaccine. The ligation must preserve the folding of the protein antigen if relevant in the case of subunit vaccines and the adjuvant properties of the ligated lipopeptide.

**Figure 13.**
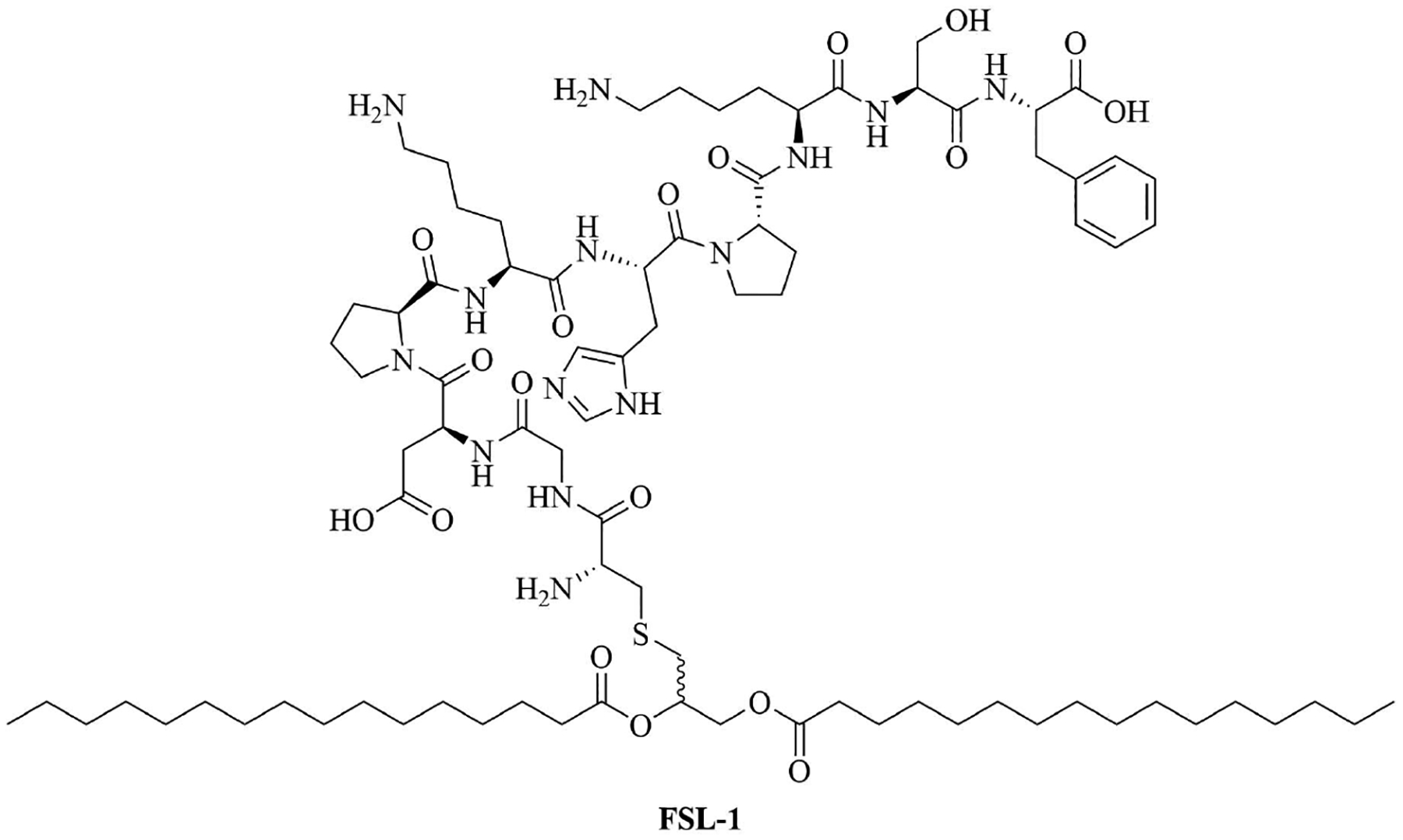
Structure of FSL-1

Sortase A ligation been used in this purpose with FSL-1 (Figure 13). Ligation of three repeats of the J14 epitope of the M protein (J14×3), a virulence factor of *S. pyogenes* enabling the bacteria to resist phagocytosis in the absence of antigen specific antibodies, and FSL-1 preserved the TLR2 agonistic character of FSL-1 and the ligation product did not elicit cell death as per evaluate by a 3-(4,5-dimethylthiazol-2-yl)-2,5-diphenyltetrazolium bromide (MTT) assay [94]. *In vivo* studies comparing free FSL-1 and protein antigen to the self-adjuvanted vaccine revealed a drastic difference in the protection against *S. pyogenes*: upon lethal challenge, mice vaccinated with the conjugate vaccine had a survival rate of 80% whereas mice vaccinated with free FSL-1 and free protein antigen only had a 30% survival rate [95]. The same free protein adjuvanted with alum only had a 40% survival rate upon lethal challenge, highlighting the importance of developing new adjuvants.

A similar strategy was used to design a self-adjuvanted vaccine against *H. pylori*. The Pam_2_CS fragment of Pam_2_CSK_4_ was covalently linked to two protein antigens of *H. pylori*, UreB (conjugate Hp4) and CagA (conjugate Hp10). Both conjugates maintained TLR2 activity and interestingly led to the upregulation of *TLR2*, *TLR4*, *NOD2*, *NLRP3*, retinoic acid-inducible gene I (*RIG-I*) and interferon-stimulated gene 15 (*ISG15*) expression on bone marrow derived DCs (BMDCs). The conjugates self-assembled into nanoparticles of 50 nm to 60 nm in diameter and were endocytosed by BMDCs. The unconjugated protein antigens were only adsorbed on the cell surface, stressing the importance of the presence of the Pam_2_CS fragment in the construct. Hp4 and Hp10 were able to elicit BMDC and macrophage maturation and treatment of BMDC with these constructs led to the secretion of IL-4, IFN-γ, and IL-17a. In comparison, treatment of BMDC with unconjugated CagA and Pam_2_CSK_4_ did not elicit the secretion of the abovementioned cytokines [80].

**Figure 14.**
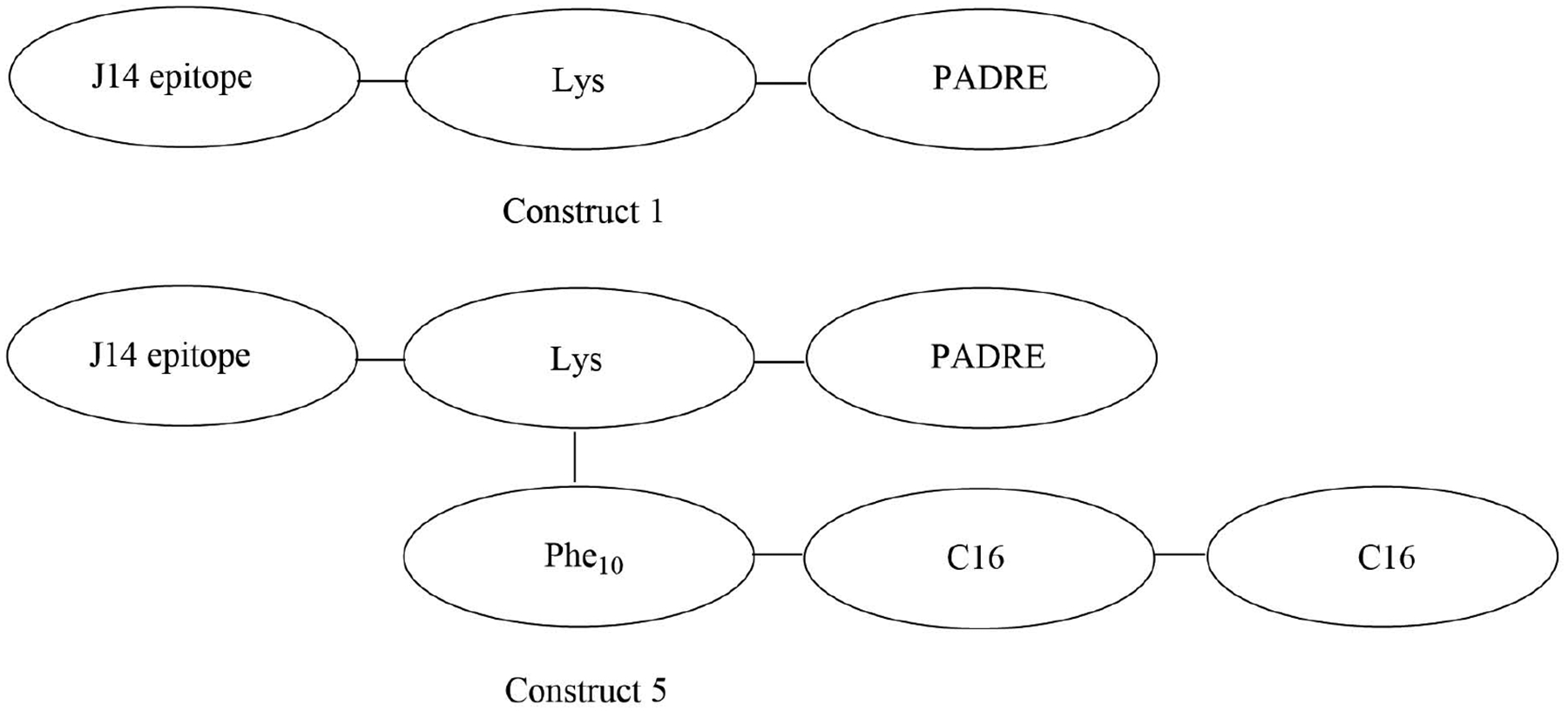
Structures of construct 1 and construct 5. PADRE: pan human leucocyte antigen DR isotype binding epitope; Phe_10_: polyphenylalanine (10 repeats); C16: 2-(*R*/*S*)-aminohexadecanoic acid

The introduction of a lipidated polyphenylalanine peptide into the vaccine construct can also lead to increased immune responses. Construct 5 (Figure 14) self-assembled into 10 nm particles with an alpha helical secondary structure, which was found to be critical in the formation of opsonizing antibodies. Self-adjuvanted construct 5 and construct 1 (Figure 14) adjuvanted with complete Freund’s adjuvant (CFA) elicited the same amount of anti-J14 specific IgG in serum, which represents up to a single-fold increase compared to the non-lipidated and mono-lipidated constructs. The increase in potency could also be due to the formation of nanoparticles of 10 nm in diameter in the case of construct 5, which falls in the optimal range for endocytosis, unlike the other constructs which only self-assembled in 5 nm particles, too small for optimal endocytosis [96].

**Figure 15.**
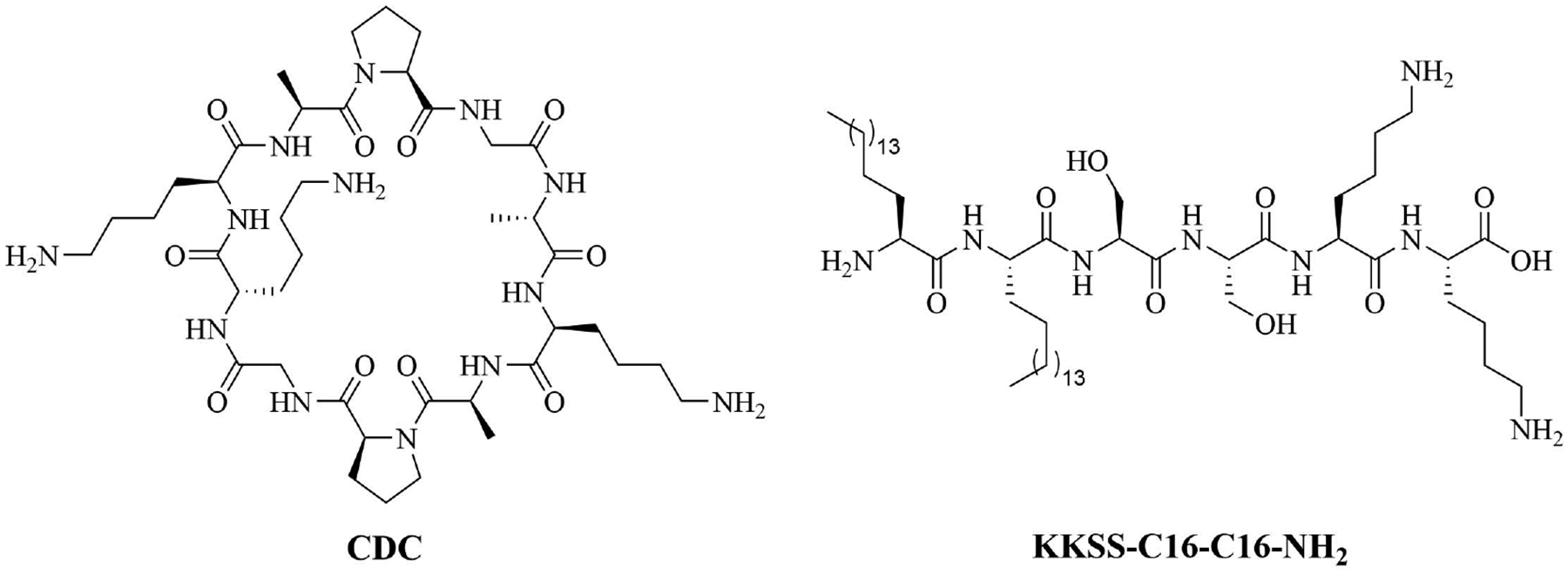
Structures of CDC and lipopeptide adjuvant KKSS-C16-C16-NH_2_

A physical mixture of antigen and adjuvants can lead to a balanced Th1/Th2 immune response, which cannot be achieved by using traditional alum adjuvants. CDC (Figure 15) was physically mixed with a lipopeptide adjuvant KKSS-C16-C16-NH_2_ (Figure 15) and J8-PADRE, a conjugate of epitope J8 and universal Th epitope PADRE, to form nanoparticles of about 857 nm, and injected into mice. This vaccine called Physical Mixture A, led to the same concentration of anti-J8 antibodies as a vaccine containing J8-PADRE and CFA. Antibody titers decreased when the antigen was conjugated to CDC and in the absence of the lipopeptide or CDC [77–79].

### Bacterial lipoproteins as a source of lipopeptide adjuvants

Bacterial lipoproteins are a source of potential lipopeptide adjuvants, as they have been shown to have TLR2 activity. They are formed by a cationic part, a hydrophobic region, and a lipobox sequence. The consensus lipobox peptide sequence [LVI][ASTVI][ASG][C] is present at the N-terminus of the lipoprotein [97]. The cysteine residue is covalently attached to diacylglycerol by lipoprotein diacylglyceryl transferase then the lipobox is cleaved, making the cysteine residue the N-terminus of the protein. The protein is subsequently lipidated to contain two or three lipid chains, binding to the TLR2/TLR6 heterodimer and the TLR2/TLR1 heterodimer, respectively [98]. Identification of bacterial lipoproteins could lead to the development of self-adjuvanted vaccines if the lipoprotein contains protective epitopes but also to the discovery of new synthetic lipopeptide adjuvants.

Vaccination against *Bordetella pertussis* (*B. pertussis*) used to be carried out with a wP vaccine. The endogenous TLR2 ligands present of the outer membrane of *B. Pertussis* provide a Th1/Th17 balanced immune response but this highly prophylactic vaccine is losing in popularity because of safety concerns to the benefit of an aP vaccine, hoped to be as efficient but safer. The acellular vaccine uses alum as an adjuvant and instead of the highly protective Th1/Th17 immune response, it gives a Th2 mediated immune response. The Th2 response is protective but less efficient as individuals vaccinated with aP are 2.2 times more likely to develop the disease [99]. Bacterial lipoprotein (BP1569) has been identified and tested for its adjuvanting abilities by Dunne et al. [100] in 2015. This lipoprotein was confirmed to be a TLR2/TLR1 ligand and induced DC maturation. From BP1569 a lipopeptide analog was synthetized: LP1569. This lipopeptide contains a triacetylated cysteine followed by the 11 N-terminus AAs of mature BP1569, giving it its cationic nature. The TLR2 character of this lipopeptide was confirmed by an *in vitro* antibody assay. Injection of mice with LP1569 induced DC maturation and pro-inflammatory IL-6 cytokine secretion in murine spleen cells. Upon co-stimulation with IL-23, γδ T cells secreted IL-17. Mice subsequently received two doses of one of three different formulations: antigens alone, with LP1569 as an adjuvant, or with alum as an adjuvant. They were then challenged with a lethal aerosol dose of *B. pertussis*. A strong Th1/Th17 immune response was observed in the vaccine containing LP1569 whereas a Th2 immune response was observed as expected in the mice vaccinated with alum. Mice vaccinated with LP1569 were the only ones to show no colonies of *B. pertussis* in the trachea 10 days after the challenge. The LP1569 vaccine was also superior to the alum one in the lungs, with the mice having 3-fold fewer colonies in the lungs with LP1569 14 days after challenge [100]. Allen et al. [101] used a synergistic adjuvant comprised of cyclic diguanylate (C-di-GMP), an agonist for intracellular receptor stimulator of interferon genes, and LP1569 for vaccination against *B. pertussis:* LP-GMP. Intranasal delivery of the vaccine adjuvanted with LP-GMP led to an over 10-fold increase in tissue resident memory T cell in the lungs and a 2-fold decrease in lung colonies, compared to the same vaccine administered intraperitoneally. The intranasal delivery of the vaccine also provided a Th1/Th17 balanced immune response whereas the mice vaccinated intraperitoneally only had a Th1 immune response. Upon challenge, intraperitoneally vaccinated mice showed a 2-fold decrease of colonies in the lungs as compared to the mice vaccinated with the same antigens but with alum as an adjuvant, after 14 days. Mice immunized with two doses of the vaccine containing LP-GMP (intranasally and intraperitoneally) and challenged 10 months after the 2nd immunization cleared the infection in the nose and lungs 14 days after the challenge, proving that LP-GMP helped sustain protection against *B. pertussis* [101].

**Figure 16.**
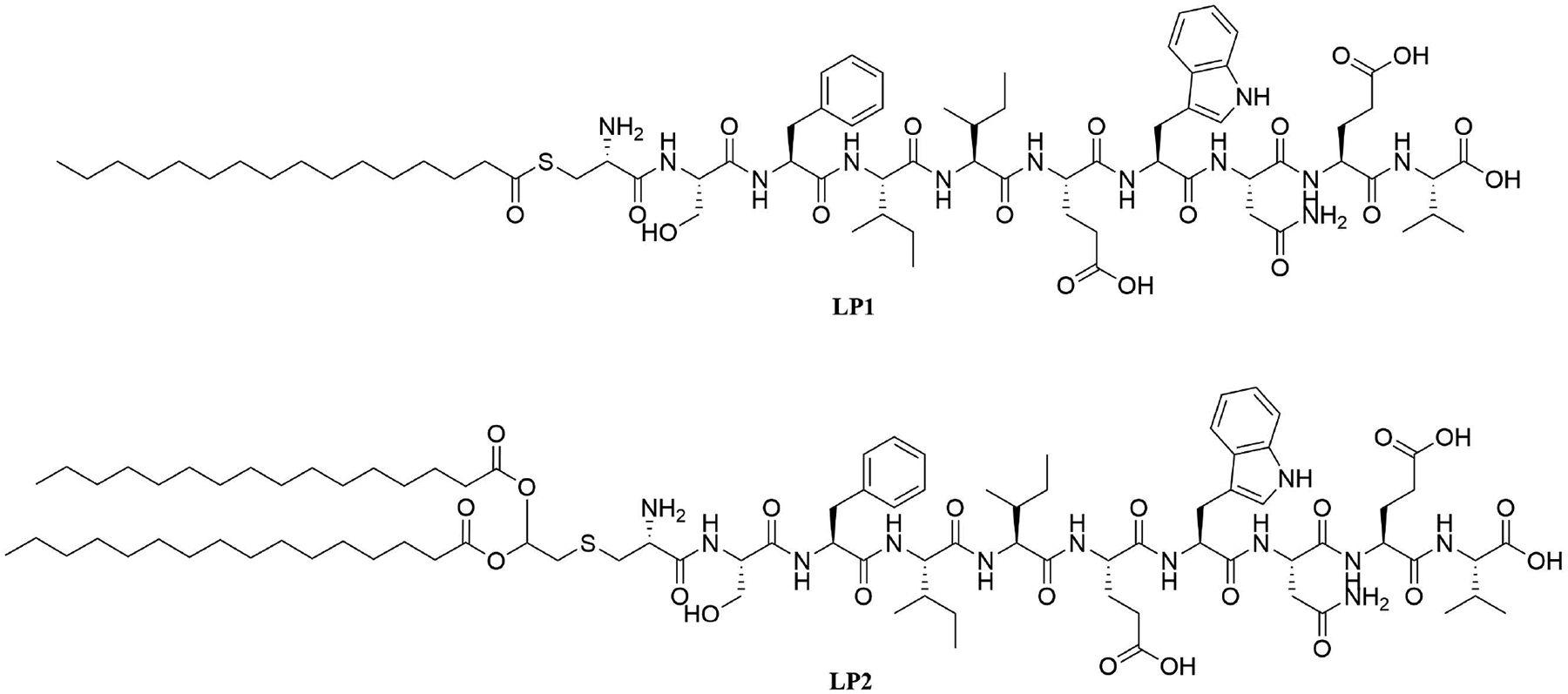
Structures of LP1 and LP2

*H. pylori* adhesin A (HpaA) is a surface lipoprotein essential for *H. pylori* gastric mucosa colonization. Non-lipidated recombinantly expressed HpaA (rHpaA) only gives weak, non-protective immune responses. To increase the immune response of a vaccine against *H. pylori* containing this protein, 2 synthetic lipopeptides LP1 and LP2 (Figure 16), respectively mono-and di-palmitoylated, comprised of the N-terminal AAs of HpaA were synthetized and their adjuvant character evaluated. Both LP1 and LP2 were proven to be TLR2 agonists *in vitro* and induced DC maturation, with LP2 being more potent. Mice immunized intranasally with rHpaA and LP2, unlike mice that received the same vaccine intramuscularly, showed a significant reduction of bacterial colonies in the stomach after challenge compared to the negative phosphate buffered saline (PBS) control group. Th1 and Th17 immune responses were involved in the protection against *H. pylori* in this assay but there was no specific immune response against LP2 [81].

## Conclusions

With the number of annual deaths due to MDR and XDR strains skyrocketing, antibiotics are not sufficient anymore to combat bacterial infections. The development of antibiotics has been very slow, as no new class of antibiotic has been approved for human use since the 1980s while the number of clinical MDR and XDR strains is increasing faster and faster [102]. Using antibiotics in combination with adjuvant is the most promising strategy to combat AMR nowadays: they allow for the repurposing of pre-existing antibiotics while limiting the surge of new MDR and XDR strains. Lipopeptides are molecules of choice for this purpose as their amphiphilic nature allows them to penetrate the lipophilic and hydrophilic barriers of GNB. Issues of stability have been addressed by utilizing peptidomimetics instead of traditional peptides, incorporating non-canonical AA or a lipid chain in the peptidyl backbone, amongst others, while maintaining low toxicity and hemolytic activity. The synergistic results of traditionally GPB reserved antibiotics used in combination with lipopeptide adjuvants against GNB are very promising and open the door to future clinical trials.

The development of new vaccines goes hand in hand with the development of new adjuvants that will allow for the immune response to be tailored to the pathogens. The array of vaccine adjuvants is not wide enough to allow for satisfying protection against current pathogens. Lipopeptides are a target of choice as they are recognized by PRR and lead to different types of immune responses. The recurring problem of in sera stability of those adjuvants has been addressed in a wide array of possibilities ranging from structure optimization to turning the adjuvants into epitope carriers, using peptidomimetics or encapsulation in liposomes. Lipopeptide as vaccine adjuvants are slowly reaching clinical trials, with Pam_2_CSK_4_ being included in a prophylactic and curative drug for pulmonary infections: PUL-042 [64]. In the context of cancer vaccination, a human volunteer has received a single injection of a vaccine containing Pam_3_CSK_4_ derivative XS15 [82]. The strong immune response observed a year after the injection, as well as the low toxicity of the vaccine is a proof of concept that will have to be further explored in clinical trials.

Lipopeptide adjuvants will require low toxicity levels, minimizing adverse effects, to be successful in clinical trials, while maintaining a high adjuvant effect. Adjuvants are not the only constructs that need careful optimization to produce an effective vaccine: epitopes play a crucial role in the immunogenicity of a vaccine [103]. Under those conditions, lipopeptides, because they are inexpensive and straightforward to synthetize and have a high potentiator effect, could offer one of the best chances of a fight against MDR and XDR pathogens.

## Figures and Tables

**Table 1.** Mechanisms of bacterial resistance

Type of resistance	Cause	Resulting phenotype
Acquired [29]	Mutations, horizontal gene transfer	Acquired antibiotic resistance in MDR and XDR strains (β-lactamase production, colistin resistance, polymyxin resistance…)
Adaptive [27, 28]	Temporary change in phenotype due to environmental causes, quickly reversible	Efflux pump upregulation
Intrinsic [30]	Resistance not caused by acquired or adaptive abilities	Outer membrane, efflux pump, biofilm

**Table 2.** Summary of mentioned lipopeptide antibiotic adjuvants

Structure	Name (abbreviation, number in text)	Source	Application
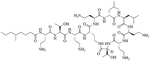	Colistin	*Paenibacillus polymyxa* subspecies *colistinus* [31]	Last resort antibiotic for GNB infections [31, 32]
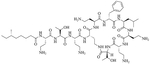	Polymyxin B	*Paenibacillus polymyxa* subspecies *colistinus* [33]	Last resort antibiotic for GNB infections [33]
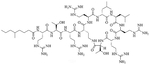	Guanidinylated colistin (GCol)	Modification of colistin [34]	Antibiotic adjuvant, synergy with rifampicin, novobiocin, and erythromycin against *Escherichia coli (E. coli), Acinetobacter baumannii (A. baumannii)*, and *P. aeruginosa* [34]
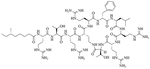	GCol polymyxin B (GPMB)	Modification of polymyxin B [34]	Antibiotic adjuvant, synergy with vancomycin, clindamycin, and linezolid against *E. coli, A. baumannii*, and *P. aeruginosa* [34]
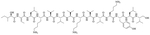	Brevibacillin 2V	*Brevibacillus laterosporus* [35, 36]	Antibiotic adjuvant, synergy with nalidixic acid, azithromycin, rifampicin, and amikacin against *E. coli, Klebsiella pneumoniae (K. pneumoniae), P. aeruginosa*, and *A. baumannii* [35, 36]
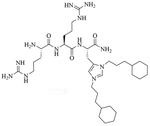	Short antimicrobial peptidomimetic 5 (SAMP-5)	Peptidomimetics	Antibiotic with activity against *E. coli* and *P. aeruginosa* [37]; antibiotic adjuvant, synergy with ciprofloxacin, oxacillin, and chloramphenicol against *P. aeruginosa* [38]; antibiotic adjuvant, synergy with ciprofloxacin and oxacillin against *E. coli* [38]
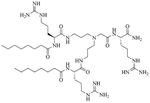	Dilipid ultrashort tetrabasic peptidomimetic (dUSTBP) di(C_8_-Arg)-Nbap-Arg-NH_2_ (8)	Peptidomimetics [39]	Antibiotic adjuvant, synergy with novobiocin and rifampicin against *P. aeruginosa*, *A. baumannii*, and *Enterobacteriaceae* [39]
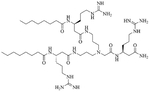	dUSTBβP 3	Modification of 8 to incorporate beta-amino acid (β-AA) instead of α-AA [40]	Antibiotic adjuvant, synergy with novobiocin and rifampicin against *P. aeruginosa*, *A. baumannii*, *K. pneumoniae*, and *Enterobacter cloacae (E. cloacae)* [40]; increased resistance against proteolysis but slower bactericidal activity than 8
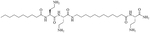	C_10_BBc_12_B	Peptidomimetics	Antibiotic adjuvant, synergy with rifampin against *P. aeruginosa*, *A. baumannii*, *K. pneumoniae*, and *E. coli*; synergy with erythromycin against *E. coli* and *K. pneumoniae*; additive effect with erythromycin against *P. aeruginosa* and *A. baumannii* [41, 42]
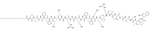	Lipo S	N/A	Used together: antibiotic adjuvant, encapsulation of ciprofloxacin with synergistic effects against *K. pneumoniae* [43]; Lipo 20 alone: antibiotic activity against *K. pneumoniae*
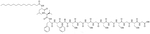	Lipo 20		

N/A: not applicable

**Table 3.** Summary of mentioned lipopeptide vaccine adjuvants

Structure	Name (abbreviation)	Source	Application
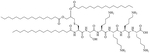	Pam_3_CSK_4_	Modification of MALP-2 (*Mycoplasma fermentas*)	Vaccine adjuvant, TLR1/TLR2 heterodimer agonist; tumor cytotoxicity [52]; used in various vaccines [53–60]
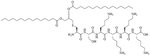	Pam_2_CSK_4_	Modification of MALP-2 (*Mycoplasma fermentas*)	Vaccine adjuvant, TLR2/TLR6 heterodimer agonist; used in various vaccines [53, 58, 61–63]; clinical trials: phase 1/2a in PUL-042, a prophylactic and therapeutic drug used against bacterial and viral pulmonary infections [64]
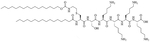	35d	Modifications of Pam_2_CSK_4_	TLR2 agonist [65]
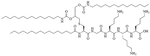	(2*S*,5*S*,8*S*,14*R*,18*R*)-2,5,8-tris(4-aminobutyl)-4,7,10,13,21-pentaoxo-14-palmitamido-18-[(tetradecylcarbamoyl)oxy]-20-oxa-16-thia-3,6,9,12,22-pentaazahexatriacontan-1-oic acid (SUP3)	Modifications of Pam_3_CSK_4_	Vaccine adjuvant (tumor model); TLR2 agonist [66]
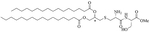	Pam_2_CS(OMe) *R*&*S*: 8a *R*: 8b *S*: 8c	Structure optimization of Pam_2_CSK_4_/Pam_3_CSK_4_ [67, 68]	Vaccine adjuvant (influenza) [68]; TLR2/6 agonist
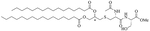	NAc-Pam_2_CS(OMe) *R*&*S*: 9a *R*: 9b *S*: 9c		TLR2/6 agonist
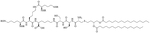	R_4_Pam_2_Cys^B^	Structure optimization of Pam_2_CSK_4_/Pam_3_CSK_4_ [69] R: 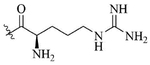	Vaccine adjuvant [ovalbumin (OVA)]; TLR2 agonist [69]
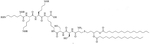	R_4_Pam_2_Cys^P^		
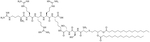	R_4_Pam_2_Cys^L^		
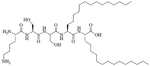	Lipid core peptide (LCP)	N/A	TLR2, self-adjuvanting antigen carrier used against group A *Streptococcus* (GAS) infections [70–72]
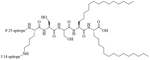	L1	Conjugation of LCP to universal Th cell epitope P25 and GAS epitope J14	Self-adjuvanting vaccine [70, 71]
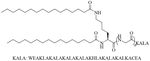	lipoKALA	N/A	Vaccine adjuvant used in combination with L1 to obtain J8-specific opsonizing antibodies upon vaccination [72, 73]
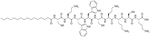	AG2-C_16_	N/A	Self-adjuvanting vaccine carrier [bovine serum albumin (BSA)] [74]
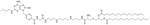	PamadiFectin	Modification of Pam_2_CSK_4_	TLR2/TLR7 agonist [75]
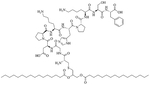	Fibroblast stimulating lipopeptide 1 (FSL-1)	*Streptococcus pyogenes* (*S*. *pyogenes*)	TLR2 agonist, vaccine adjuvant used against GAS [76]
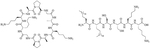	Cyclic decapeptide carrier (CDC) and lipopeptide adjuvant KKSS-C16-C16-NH_2_	N/A	Vaccine adjuvant system used against GAS [77–79]
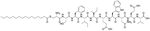	LP1	*Helicobacter pylori* (*H. pylori*) adhesin A	TLR2 agonist, vaccine adjuvant against *H. pylori* [80, 81]
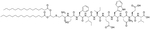	LP2		

## Abbreviations

*A. baumannii*: *Acinetobacter baumannii*
AMPs: antimicrobial peptides
AMR: antimicrobial resistance
aP: acellular *pertussis*
APC: antigen-presenting cell
*B. pertussis*: *Bordetella pertussis*
BMDCs: bone marrow derived dendritic cells
BSA: bovine serum albumin
CD40: clusters of differentiation 40
CDC: cyclic decapeptide carrier
CpG: cytosine phosphoguanine
CPPs: cell penetrating peptides
DC: dendritic cell
*E. coli*: *Escherichia coli*
FDA: Food and Drug Administration
FICI: fraction inhibitory concentration index
FSL-1: fibroblast stimulating lipopeptide 1
GAS: group A *Streptococcus*
GCol: guanidinylated colistin
GNB: gram-negative bacteria
GPB: gram-positive bacteria
GPMB: guanidinylated colistin polymyxin B
*H. pylori*: *Helicobacter pylori*
HpaA: *Helicobacter pylori* adhesin A
IgG: immunoglobulin G
IL-6: interleukin 6
*K. pneumoniae*: *Klebsiella pneumoniae*
LCP: lipid core peptide
Lys: lysine
MDP: muramyl dipeptide
MDR: multidrug-resistant
MIC: minimum inhibitory concentration
NOD2: nucleotide-binding oligomerization domain 2
OVA: ovalbumin
*P. aeruginosa*: *Pseudomonas aeruginosa*
PAc: protein antigen c
PADRE: pan human leucocyte antigen DR isotype binding epitope
Pam_2_CSK_4_: *S*-[2,3-bis(palmitoyloxy)propyl]cysteinyl-seryl(lysyl)_3_-Lys
Pam_3_CSK_4_: *N*-palmitoyl-*S*-[2,3-bis(palmitoyloxy)propyl]cysteinyl-seryl(lysyl)_3_-Lys
PLA: polylactic acid
PRRs: pattern recognition receptors
*S. pyogenes*: *Streptococcus pyogenes*
SAMP-5: short antimicrobial peptidomimetic 5
SLP: synthetically long peptide
Th1: type 1 T helper cell
TLRs: Toll-like receptors
TNF-α: tumor necrosis factor alpha
USD: US dollars
wP: whole-cell *pertussis*
XDR: extensively drug-resistant
β-AA: beta-amino acid
